# Steps towards quality of open access publishing

**DOI:** 10.31138/mjr.29.4.184

**Published:** 2018-12-18

**Authors:** Armen Yuri Gasparyan, George D. Kitas

**Affiliations:** 1 Departments of Rheumatology and Research and Development, Dudley Group NHS Foundation Trust (Teaching Trust of the University of Birmingham, UK), Russells Hall Hospital, Dudley, West Midlands, UK;; 2 Arthritis Research UK Epidemiology Unit, University of Manchester, Manchester, UK

**Keywords:** digital tools, periodicals as topic, publishing, quality control, open access, rheumatology

In times of information deluge, editing and publishing a scholarly journal is increasingly dependent on digital tools and artificial intelligence.^1^ To a large extent, this is a positive trend which drives mass proliferation of scholarly articles and online journals across academic disciplines. Rheumatology as a research-intensive discipline may particularly benefit from the emerging digital evaluation tools, systematic search engines, distribution platforms, and repositories. Currently, processing journal submissions through advanced online systems allows detecting major inconsistencies and mistakes in research reporting and formatting manuscripts. Such systems, linked to regularly updated reviewer instructions of global editorial associations and supported by external bibliographic searches, enable fast evaluation of the novelty, originality, methodological rigour, and implications of the manuscripts by peer reviewers.

The author and reviewer identifiers, and particularly the *Open Researcher and Contributor IDs* (ORCID; https://orcid.org/ ), are now employed by most publishers to validate individual profiles at the peer review and increase visibility of their contributions post-publication. The *Mediterranean Journal of Rheumatology* has also encouraged its contributors to register with and submit informative ORCID iDs with biographical notes, publication lists, and other academic accomplishments. The ORCID iDs are now integrated with the the *Publons* platform ( https://publons.com ), yet another indispensable digital tool for crediting reviewers and selecting active and skilled experts for further evaluations.^2,3^ Notably, the number of academic contributors with ORCID iDs is fast approaching the level of 6 million, and more publishers and standalone journals are partnering with Publons to streamline the peer review activities and global recognition of their reviewers. With the integration of Publons with Web of Science (WoS, Clarivate Analytics) in 2017 and further upgrades of the platform, the registered scholars are now offered automatic updates of their profiles by listing their WoS-indexed articles and tracking related citations. Publons profiles are particularly valued by emerging scholarly journals, aiming to attract scarcely available reviewers and increase the quality of peer review.^4,5^ The total number of registered reviewers on Publons is 541,093, including 571 in rheumatology (as of December 19, 2018). The *Mediterranean Journal of Rheumatology* acquired its profile on Publons this year to increase transparency of the peer review activities and give deserved academic credits to its best contributors ( https://publons.com/journal/101214/mediterranean-journal-of-rheumatology ).

The speed of processing and visibility of scholarly contributions are also increasingly dependent on Digital Object Identifiers (DOIs) issued by *Crossref*, a powerful system for cross-linking references and updating individual digital profiles (http://crossref.org/). Launched in 2000, Crossref is currently run by the Publishers International Linking Association. The reference validation and formatting tools supported by Crossref permanent identifiers guarantee the accuracy of technical editing, formatting, and detecting plagiarism, to name just a few advantages of the system. With the expansion of Crossref services and integration with journal platforms (i.e., Open Journal System of the Public Knowledge Project), ensuring the efficiency of online publishing, navigating through myriads of scientific articles, and indexing with bibliographic databases are becoming closely interconnected. Once and for all, the concept of discoverability of scholarly items and systematic searches through online databases has shifted towards permanent article identifiers.^6,7^ Given the growing importance of the Crossref services, a decision was made to acquire DOIs for the *Mediterranean Journal of Rheumatology* this year to benefit from vast opportunities of improved visibility and integration with digital platforms and databases.

Over the past two decades, open-access publishing has increased global visibility and international recognition of journals across numerous subject categories. Various open access models have increased article views, downloads, and citations, transforming all these benefits into the scientific prestige of emerging and research-intensive fields of science.^8^ The absolute majority of open-access medical journals have benefited from archiving by PubMed Central, the largest digital repository of medical articles. The editors’ relentless efforts aimed to archive the *Mediterranean Journal of Rheumatology* by PubMed Central will widen prospects of indexing for this rapidly developing periodical.

The basic principles of Open Access are outlined in two seminal documents, which are consulted by the editors of the *Mediterranean Journal of Rheumatology*, and related links are embedded in the journal instructions: the Budapest Open Access Initiative (2002) and the Berlin Declaration on Open Access (2003).^9,10^ In line with the statements of these documents, the Open Access initiative is primarily aimed to increase the use of published items for research and education.^11^ Additionally, the improved visibility, one of the main achievements of the initiative, has enabled detecting and ‘cleaning’ erroneous and fraudulent literature.^12^ Although the main attribute of Open Access is free access to published works for readers, there are other, not less important, components related to digitization, preservation, and archiving. To meet the requirements of Open Access and satisfy indexing criteria of most bibliographic services, medical journal editors should familiarize themselves with the Journal Article Tag Suite (JATS) format of Extensible Markup Language (XML), which is employed by the National Library of Medicine of the US for encoding PubMed Central-archived items.^13^ The XML format enables machine-readability and makes it possible to index quality journals by most prestigious bibliographic databases and digital repositories.

While Open Access is gaining momentum and numerous journals opt for different models of open-access publishing (i.e., gold, platinum, hybrid, green), several global initiatives have been launched to distinguish periodicals with reliable peer-reviewed contents from spurious online platforms of wasteful publishing. The Directory of Open Access Journals (DOAJ) is one such initiative with strict scientific and technical criteria, listing 12,397 open access journals from 129 countries and offering records of 3,600,965 for systematic searches (as of December 19, 2018). The number of the listed medical journals is 699, with 472,913 linked items available for searches. The latest incorporation of the *Mediterranean Journal of Rheumatology* to the area of medicine of the registry is an exemplary achievement for the whole field of rheumatology (December 10, 2018).

Launched in 2003, the DOAJ project has passed a long way and become a ‘whitelist’ of reliable periodicals. The DOAJ collaborated with several global editorial associations to set principles of transparency in publishing.^14^ The declared by the *Mediterranean Journal of Rheumatology* adherence to these principles is essential for its development and ethical promotion.

Acquiring the seal of DOAJ is a milestone in the journal digitization and recognition by the scientific community.^15,16^ For medical journals, the DOAJ listing is proposed as one of the requirements for indexing by PubMed and archiving by PubMed Central.^17^ In 2015, the Scopus database introduced the open access indicator for separate marking DOAJ-listed journals, making it easier to navigate and search through the growing number of open-access periodicals which passed stringent quality checks.^18^ Notably, the SCImago Journal & Country Rank platform currently ranks 34,171 Scopus-indexed periodicals across all subject categories, including 4,504 for open access. In the field of rheumatology there are 60 Scopus-indexed periodicals, with 20 registered with DOAJ (https://www.scimagojr.com/journalrank.php?-category=2745). These numbers will definitely continue to grow in the future.

While the recent digital innovations enormously influence the speed of publishing, editorial credentials still remain precious assets for the quality of publishing and impacting science. The journal editors with connections to global and regional editorial associations are capable of establishing networks of skilled peers, share experience, and add to the scientific prestige of respective periodicals. In the field of medicine, the *International Committee of Medical Journal Editors* (ICMJE, http://icmje.org) is perhaps the most important association; offering frequently updated editorial guidance for authors, reviewers, and editors. Editors claiming adherence to the ICMJE recommendations and enforcing its points in their daily practice offer good service to their community of authors and readers. Exemplary, the editors of the *Mediterranean Journal of Rheumatology* registered their interest in the recommendations at the ICMJE website in 2017.^19^

Several other global associations also allow advancing scientific and publishing editors’ skills.^20^ The editorial credentials and reviewer responsibilities are currently highlighted in the documents published by the *Committee on Publication Ethics*, the largest association of editors with more than 12,000 members. Member-editors of the COPE are now offered a set of updated recommendations about authorship problems, conflict of interest disclosures, ethics approval, copyright issues, journal management, and retractions.^21^ The latest incorporation of the updated publication ethics points into the instructions of the *Mediterranean Journal of Rheumatology* is an exemplary move towards better quality control for disseminating reliable information for the respective regional community.
